# Effects of Laser Machining Aluminum Alloy in Different Media

**DOI:** 10.3390/mi13071130

**Published:** 2022-07-17

**Authors:** Xiang Li, Shan Huang, Jianping Tang, Weihao Mu, Xin Xu, Xuehui Chen

**Affiliations:** 1 School of Mechanical Engineering, Hefei University of Technology, No. 193, Tunxi Road, Hefei 230009, China; lix1117@163.com; 2 School of Mechanical and Electrical Engineering, Anhui Jianzhu University, No. 292, Ziyun Road, Hefei 230601, China; hs18556919674@163.com (S.H.); muweihao@yeah.net (W.M.); 3 Hefei Fuhuang Junda Hi-Tech Information Technology Co., Ltd., No. 10, Tiantong Road, Hefei 235200, China; xinxu1121@163.com

**Keywords:** laser processing, air, water, aluminum alloy

## Abstract

To study the effects of aluminum alloys processed by a laser in air and water and at different water velocities, corresponding experiments were conducted and the impacting effects of different water velocities on the surface of the workpiece were simulated, respectively. The results show that when laser processing aluminum alloy materials in air, there is more slag and a recondensation layer on both sides of the groove, the heat-affected zone is larger and the surface processing quality is poor. When laser processing aluminum alloy materials in water, the processing quality is improved. With the increase in water velocity, the impacting and cooling effect is enhanced, the groove depth and groove width show a trend of first increasing and then decreasing, the slag and recondensation layer on both sides of the groove are reduced, the heat-affected zone is reduced and the processing quality of the groove is improved. When the water velocity reaches 30 m/s, a better groove can be obtained. Laser processing aluminum alloy materials in water can obtain better processing quality than laser processing in air.

## 1. Introduction

Aluminum alloys have been widely used in the aerospace and medical fields, with the advantages of high strength, strong corrosion resistance, easy forming and excellent mechanical properties [1,2,3]. However, conventional machining methods show difficulty in machining aluminum alloys due to their metallic properties. Moreover, deformation easily occurs in the process of machining aluminum alloys, which not only seriously affects the machining quality but also affects the tool life [4,5]. Laser technology can process metal and non-metal materials; it belongs to non-contact processing and will not cause mechanical extrusion and mechanical stress to the material. It has been widely used in various industries, with the advantages of high processing efficiency and a wide processing range [6,7]. Affected by the material properties of aluminum alloys and the effect of laser heat, there are phenomena such as a large heat-affected zone and slag accumulation during laser processing [8]. In order to reduce the defects existing in laser processing, researchers have introduced liquids in laser processing to effectively reduce or eliminate defects, and a large number of studies have been performed [9].

Cao et al. [10] studied the effects of laser power, laser repetition frequency and feed rate on the shape of the groove in the water-guided laser processing of 6061 aluminum. Studies have found that higher laser power and lower laser repetition frequency are beneficial to the formation of trenches with a vertical sidewall. Adelmann et al. [11] studied the influence of laser power on the depth of grooves in the water-guided laser processing of various materials and realized the processing of deep grooves with an aspect ratio of 1:66. Qiao et al. [12] used water-guided laser technology to successfully process a hydrophobic micro-texture on the surface of stainless steel. Huang et al. [13] proposed a non-uniform electric field-based deflected water beam guiding laser method for the water–light coupling process. The principle of the related technology was analyzed and the non-uniform electric field deflecting the water beam was simulated, which provides theoretical support for water-guided laser technology based on the non-uniform electric field deflecting the water beam. Zhang et al. [14] simulated the process of water-guided laser processing for carbon fiber-reinforced plastics. It was found that adjusting the duty cycle of the laser pulse can significantly affect the shape and temperature distribution of the composite material after drilling. Meanwhile, the experimental results show that water-guided laser processing can obtain better processing results than traditional laser processing.

Feng et al. [15] used underwater laser micro-milling technology to successfully process a microchannel with a width of 200 μm, a depth of 700 μm and a bottom roughness of 1 μm on FG-Al substrates without thermal effects. Shin et al. [16] successfully performed underwater fiber laser cutting for 50 and 60 mm thick stainless steel plates and the width of the cut was very narrow. Charee et al. [17] studied the influence of water temperature on the width and depth of the groove in the underwater nanosecond pulse laser processing of monocrystalline silicon. The study found that the use of a high water temperature can increase the material removal rate and the groove aspect ratio. Soliman [18] studied the effect of laser processing on stainless steel in water and hexane. It was found that different liquid media cause differences in roughness on the ablated stainless steel surface, and the lower surface roughness can be achieved when stainless steel is processed underwater. Long et al. [19] studied the influence mechanism of the thickness of the water layer on the etching quality of the workpiece. The results show that the initial laser absorption and the formation of cavitation bubbles after laser incidence are not affected by the immersion depth, which provides theoretical support for the choice of water layer thickness in underwater laser processing. Lv et al. [20] used nanosecond pulsed laser processing for Inconel 718 samples in air and water. It was found that the edges of the holes processed in air experienced obvious melt recast and redeposition, while the edges of the holes processed underwater were significantly smoother and there was almost no recast or redeposition.

Guo et al. [21] investigated the influence of different processing parameters on the ablation depth, width and material removal rate in water-assisted pulsed laser processing, and successfully processed micro-groove arrays on CVD diamond-coated tools. Wang et al. [22] studied the effects of laser pulse energy, water jet pressure and laser moving speed on groove depth and surface micro-morphology in the water jet-assisted laser processing of silicon nitride. It was found that the laser pulse energy and water jet pressure dominated the groove depth and microscopic morphology. Zhu et al. [23] established a numerical model of heat transfer and material ablation in the water jet-assisted laser processing of single-crystal germanium to prove that the shielding effect of laser-induced plasma increases with laser pulse energy. The water jet can not only wash away the material softened by the laser, but also effectively remove the heat accumulation in the workpiece to minimize thermal damage. It was found that the groove depth increases with the laser pulse energy, and the pressure of the water jet lowers the threshold workpiece temperature for material removal. Zhou et al. [24] analyzed the effects of heat transfer, water jet and water film in jet-assisted laser processing. It was found that the jet velocity and the shape of the water layer will affect the laser ablation efficiency and the quality of the ablation characteristics. Wang et al. [25] performed a water jet-assisted laser processing microchannel experiment on a stainless steel surface and used the response surface method to study the influence of various process parameters on the microchannel processing quality and heat-affected zone. 

Many scholars have studied the laser–water processing technology. However, there are few studies on the effects of different water velocities on the aluminum alloy processing morphology. Therefore, aluminum alloy was selected as the research object to study the influence of laser processing in air and water and analyze the influence of water velocity on the contour, width and roughness of the groove.

## 2. Laser–Water Composite Machining Model and Theoretical Analysis

**Figure 1:**
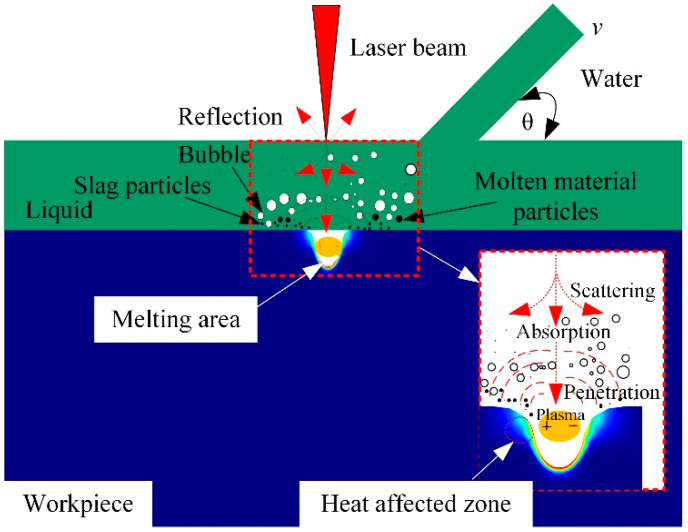
Schematic diagram of laser–water composite processing.

Laser–water composite processing combines laser processing and water jet processing technology. The impact effect of the water jet can wash away the slag and debris generated during laser processing; the cooling effect of the water jet can absorb excess laser energy, cool the processing area and reduce ablation [26]. By using the impact and cooling effect of a water jet, the processing quality is improved to a certain extent. The principle of laser–water composite processing is shown in Figure 1.

In laser–water composite processing, the water cools the processing area—that is, the convective heat exchange between the water and the laser energy. The Nusselt number is used to judge the intensity of convective heat transfer. According to the literature [27], the Nusselt number of the circular cross-section nozzle is

(1) $$N=P_{r}^{0.42}\cdot G\left(A_{r},\frac{H}{D}\right)\left[2R_{e}^{0.5}{\left(1+0.005R_{e}^{0.5}\right)}^{0.5}\right]$$

Here,

$$G=2A_{r}^{0.5}\cdot \frac{1-2.2A_{r}^{0.5}}{1+0.2A_{r}^{0.5}\cdot \left(\frac{H}{D}-6\right)};P_{r}=\frac{c\mu }{4r^{2}};A_{r}=\frac{D}{4r^{2}};\;R_{e}=\frac{\rho vl}{\mu }$$

The convective heat transfer coefficient *h* is

(2) $$h=\frac{N\cdot K}{l}$$

(3) $$h={\left(\frac{c\mu }{4r^{2}}\right)}^{0.42}\cdot 2{\left(\frac{D}{4r^{2}}\right)}^{0.5}\cdot \frac{1-2.2{\left(\frac{D}{4r^{2}}\right)}^{0.5}}{1+0.2{\left(\frac{D}{4r^{2}}\right)}^{0.5}\cdot \left(\frac{H}{D}-6\right)}\cdot \left[2{\left(\frac{\rho vl}{\mu }\right)}^{0.5}{\left(1+0.005{\left(\frac{\rho vl}{\mu }\right)}^{0.5}\right)}^{0.5}\right]\cdot K\cdot l^{-1}$$

Here, *R* is the Reynolds number; *K* is the heat transfer coefficient; *v* and *l* are the flow rate of water and the characteristic length of the processed material respectively; *μ* is the dynamic viscosity coefficient of water; *P_r_* is the Planck number of water; *D* is the nozzle diameter; *H* is the distance between the nozzle and the impact point.

It can be seen from Equation (3) that the convection heat transfer coefficient *h* is related to *H*/*D*, *Re*, *k*, *L* and *v*. Assuming that the thermal conductivity of the aluminum alloy remains unchanged, the length of the material remains unchanged, and the *H*/*D* remains unchanged, then the convective heat transfer coefficient is related to the water velocity. The convective heat transfer coefficient increases with the increase in water velocity. The laser processing area is cooled by increasing the convective heat transfer coefficient.

In laser–water composite processing, when the liquid water impacts the processing area, the water produces an impact on the material, which can wash away the slag generated after laser processing in time and improve the surface quality of the workpiece. The impact of the water jet on the surface of the workpiece is divided into two stages: the water hammer pressure and the stagnation pressure stage. In the first stage, the water jet impacts the workpiece and generates a shock wave. The compressed water acts on the workpiece as a “water hammer” to generate pressure. In the second stage, after the first stage, a stable impinging jet is formed. At this time, the center pressure of the jet will gradually oscillate and decrease, forming a stable stagnation pressure. Then, the water jet will continuously impact the workpiece at the same speed and the dynamic pressure of the jet center will also stabilize at the stagnation pressure. According to the Bernoulli equation, the water hammer pressure and stagnation pressure can be obtained [28]:

(4) $$p_{w}=\rho kv^{2}+\rho vc_{0}$$

(5) $$p_{s}=\frac{1}{2}\rho v^{2}$$

Here, *p_w_* represents the water hammer pressure; *p_s_* represents the stagnation pressure; *ρ* is the density of water; v is the jet velocity; *c*_0_ is the shock wave velocity of water; *k* is the coefficient (approximately 2 when the jet velocity is less than 1000 m/s). As the release wave propagates from the periphery to the center position, the impacting pressure quickly decreases to the stagnation pressure and remains essentially stable. Under the action of a continuous and stable jet, the stagnation pressure is equal to the central dynamic pressure of the water jet.

It can be seen from Equation (5) that the force in the jet direction gradually increases with the increase in water velocity, which can flush away the molten material after laser processing and reduce the accumulation of slag. However, the higher water velocity in actual processing not only puts forward higher requirements on the clamping of the workpiece and the water jet processing system, but also produces water mist, shields the laser and affects the laser focus radius.

## 3. Experimental Research

**Figure 2:**
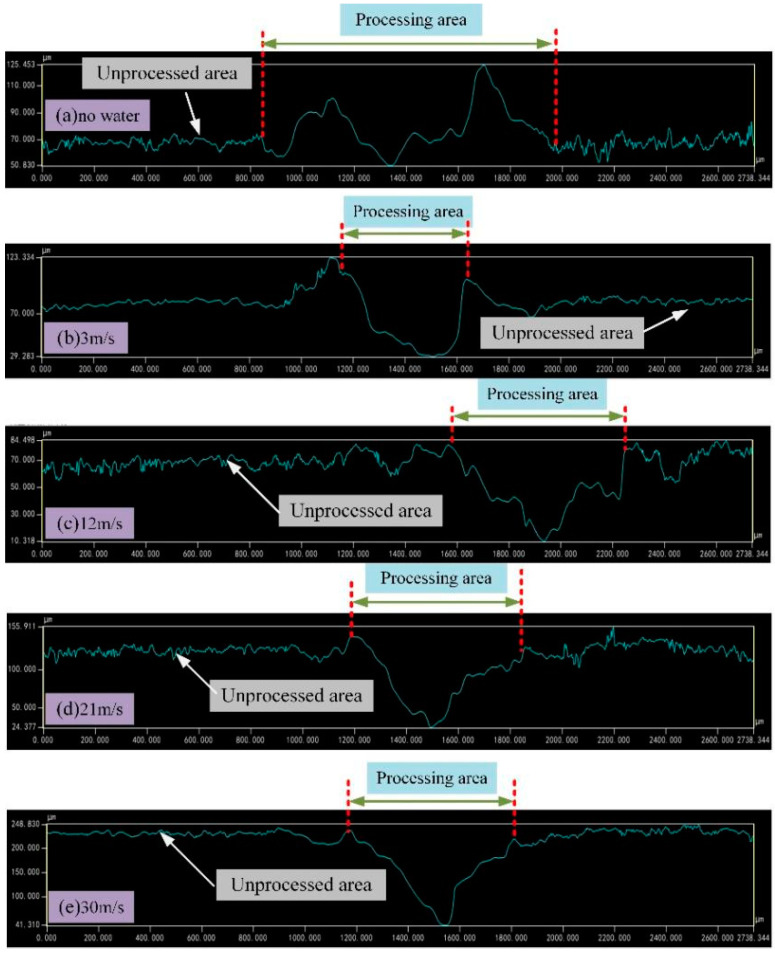
Groove profile.

**Figure 3:**
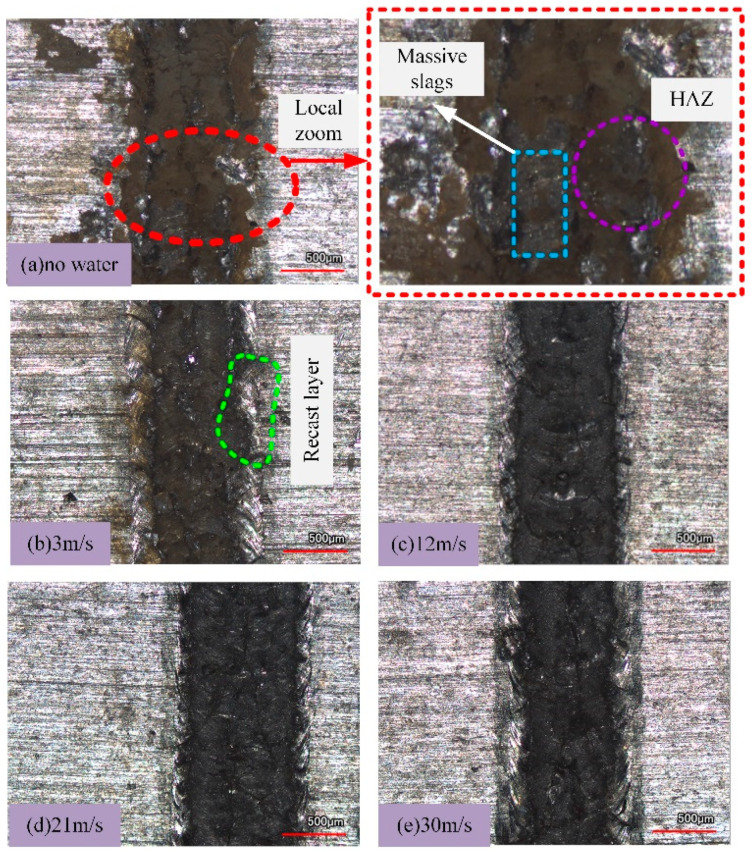
Groove width.

**Figure 4:**
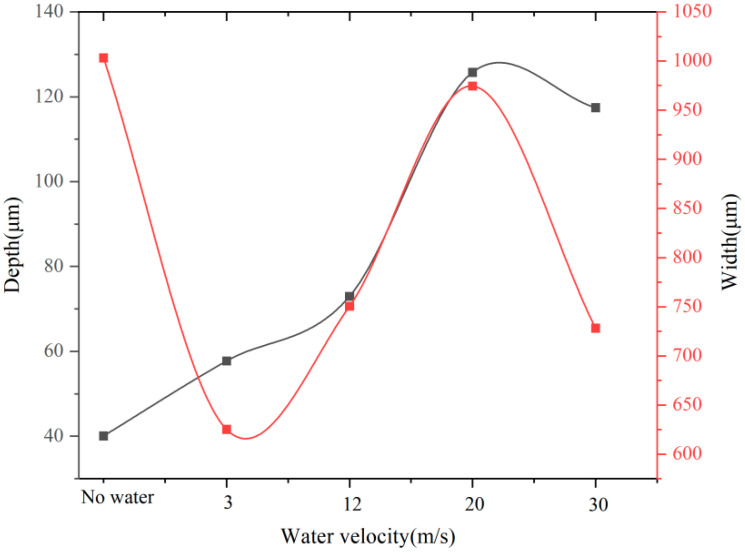
Variation trend of depth and width.

**Figure 5:**
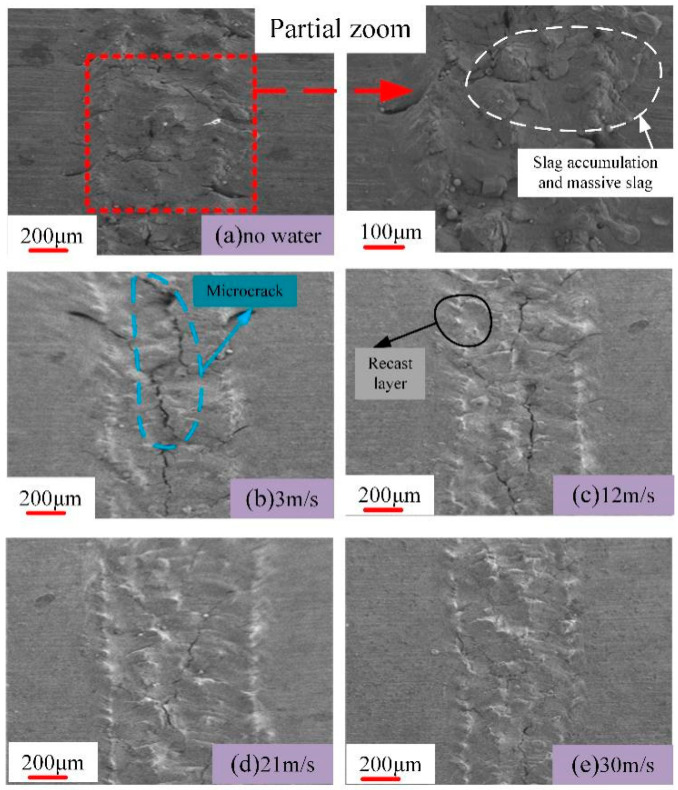
Groove micro-morphology.

Aluminum alloy was selected as the research object. The TY-LFM-500 multifunctional laser etching machine was selected (max. power 500 W, pulse frequency 1–500 Hz, pulse width 0.1–20 ms, wavelength 1064 nm). The processing parameters were set as follows: laser current 125 A, laser pulse width 3 ms, repetition frequency 10 Hz, laser scanning speed 1.5 mm/s, circular spot diameter 1 mm, water jet angle 60°and water jet velocities are 3 m/s, 12 m/s, 21 m/s and 30 m/s. Absolute ethanol was used to clean the 7075 aluminum alloy surface before laser machining. The results of laser processing and laser–water composite processing are shown in Figure 2, Figure 3, Figure 4 and Figure 5 (Figure 2 is the cross-sectional profile of the groove; Figure 3 is the width; Figure 4 is the variation trend of depth and width; Figure 5 is the micro-topography).

### 3.1. The Influence of the Medium on the Processing Result

Comparing Figure 2, Figure 3, Figure 4 and Figure 5, it can be seen that when laser processing aluminum alloy in air, the depth and width of the groove are greater than those obtained by laser processing in water. However, more recondensed layers are produced on both sides of the groove, a large amount of slag is accumulated on the bottom of the groove and the ablation phenomenon is more serious.

It was observed that during laser processing in air, the laser beam directly irradiates the surface of the aluminum alloy material and the light energy is converted into heat energy and conducted into the material. The aluminum alloy material absorbs a large amount of laser energy to reach the material ablation threshold, and the processed material is blown away under the action of the auxiliary gas. The blow-off ability of the auxiliary gas is limited and it cannot blow a large amount of molten material away from the processing area in time. The molten material rapidly cools and solidifies in the processing area and accumulates in the groove, which will further transfer heat to the inside of the material, causing excessive thermal stress inside the material to produce cracks.

Although there is still slag that accumulates on the bottom and sides of the groove when the water velocity is 3 m/s, the processed surface is no longer similar to that of laser processing in air, as the slag and recast layer are significantly reduced.

In laser–water composite processing, the water has the effects of impact and cooling. The cooling action of the water jet absorbs excess laser energy applied to the surface of the material, which reduces the heat-affected zone and ablation. The impact of the water can wash away the slag generated by the laser action material from the processing area in time, which causes the accumulation of slag and the formation of the re-condensed layer to be reduced, and the processing quality is improved. When the water velocity is small, the convection heat exchange between the water and the laser is strong. Although the water also has an impact on the material, the convective heat exchange between the water jet and the laser dominates, and a large amount of laser energy is absorbed by the water, so the depth of the groove is small. As the water velocity increases, both the water–laser–material impact and cooling effects increase. Although there is strong convective heat exchange between the water jet and the laser, the force of the impact material increases greatly due to the high speed of the water jet. At this time, the impact of the water jet is dominant, and the molten slag after the action of the laser can be punched out of the groove in time to reduce the absorption of laser energy by the slag, thereby reducing the absorption of laser energy by the slag, so the depth of the groove is small. As the water velocity continues to increase, the depth of the groove decreases. At this time, the impact of water on the material is greater, and there is strong convective heat exchange between the water and the laser; the experimental device shakes more seriously at this time due to the high speed of the water. When the water impacts the material, the impact center of the jet is deviated. When it impacts the surface of the material, the water beam diverges greatly, which affects the laser focus spot, and the laser energy loss is large, so the depth of the groove is small.

The convection heat exchange between liquid–material–laser is relatively strong and laser energy loss is greater [29]. The actual energy of the laser irradiated on the surface of the material is smaller than the laser energy in air. Therefore, the groove depth and width with the laser processing of aluminum alloy materials in water is smaller than that with laser processing in air. The water is instantly vaporized under the high-temperature action of the laser, forming cavitation bubbles in the water accumulated in the groove [30]. During the collapse stage of the cavitation bubble, shock waves and micro-jets will be formed to act in the groove. The shock wave prevents the molten aluminum alloy from flowing to the bottom of the groove. The high-pressure shock wave causes the melt to sputter from the bottom of the groove to the notch and solidify into a small amount of massive slag on the groove wall and the incision [31].

### 3.2. Effect of Water Velocity on Processing Results

It can be seen from Figure 2, Figure 3, Figure 4 and Figure 5 that when the water velocity is 3 m/s, a small amount of unremoved slag still exists in the groove, and the depth and width of the groove are small. It was observed that the convective heat transfer between the laser beam and water is stronger and the laser loses more energy. At the same time, the impact of water on the groove is relatively small, and thus cannot completely wash away the products after laser processing. The cooling effect is dominant in this process, resulting in relatively small depth and width.

As the water velocity increases, the groove depth and width gradually increase, the slag is washed away and the processing quality is improved. It was observed that the increase in water velocity will increase the impact force acting on the material, which can wash away the slag generated in time. However, when the water velocity increases to 30 m/s, the depth and width of the groove decrease instead. It was posited that when the water velocity is high, although the impact effect on the material is enhanced, the convective heat transfer between the laser–material–water is also enhanced at this time. Moreover, the high water velocity will cause the nozzle device to vibrate violently, causing the jet center to deviate from the impacting center, and the molten aluminum alloy cannot be flushed out of the groove in time. Meanwhile, the impact effect of water is weaker than the cooling effect, the laser energy acting on the material is greatly reduced, and the groove depth and width are relatively reduced [32].

**Figure 6:**
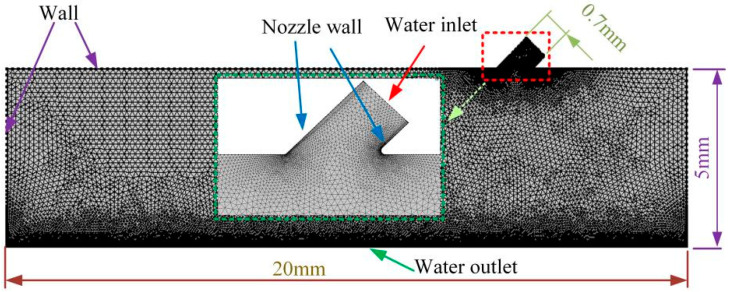
Two-dimensional model and meshing.

**Figure 7:**
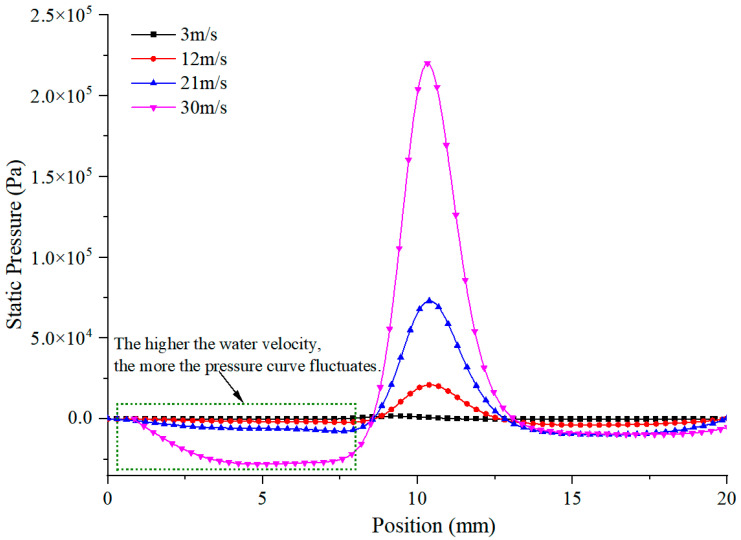
Impact force generated by different water velocities hitting the workpiece.

In order to analyze the impact effect of different water velocities on the workpiece, finite element software was used to simulate the impacting force generated when the workpiece is impacted by different water velocities. In the experiment, the diameter of the nozzle was 0.7 mm, and the distance between the nozzle and the machining surface of the workpiece was 5 mm. The model was simplified to a two-dimensional model and meshed, as shown in Figure 6. The water inlet, nozzle wall and water outlet were named separately in the software, and the water and the material surface contact part were chamfered for the purpose of reducing the effect of stress concentration on the simulation results. Different water inlet velocities were set in the software, such as 3 m/s, 12 m/s, 21 m/s, 30 m/s. The outlet pressure was set to a standard atmospheric pressure of 0.1 MPa and a non-slip wall was used. The k-ε standard model was adopted as the turbulence model and the solver adopted the SIPMLE algorithm. The pressure curve of the impact surface was extracted, as shown in Figure 7.

It can be seen from Figure 7 that when the water jet velocity is 3 m/s, the impact force generated on the surface of the acting material is small and it has almost no effect on the removal of the material. As the water velocity increases, the impact force gradually increases and the increase gradient is larger.

It can be seen from the magnified area in Figure 7 that with the increase in the water velocity, the water flow fluctuates greatly, which interferes with the laser beam and affects the processing results. When the water velocity is 3 m/s, the water flow is relatively stable and the fluctuation is small, while the interference to the laser beam is weak. When the water velocity increases to 30 m/s, the water acting on the workpiece fluctuates greatly. At this time, the water beam diverges seriously and the actual laser energy of the irradiated material is affected due to the increased refraction or reflection effect of the “water mist” generated by the larger water jet velocity on the laser beam, which will affect the processing results.

From the point of view of material removal, the higher the water velocity, the more favorable the actual processing is when the actual conditions are allowed. However, it can be seen from Figure 2e, Figure 3e and Figure 7 that when the water velocity is high, such as 30 m/s, the water fluctuates greatly on the surface of the material and the water beam diverges, which is not conducive to processing.

**Figure 8:**
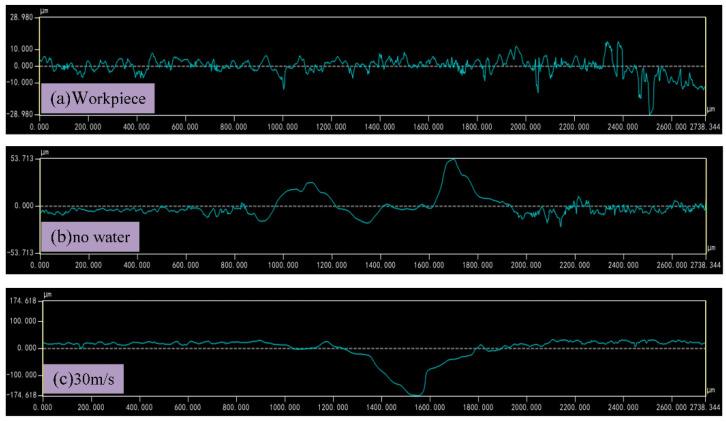
Roughness before and after laser processing.

The roughness of laser-machined aluminum alloys in air and water was compared, as shown in Figure 8. Figure 8a shows the result before processing, Figure 8b shows the result of laser processing in air, and Figure 8c shows the result when the water velocity is 30 m/s.

It can be seen from Figure 8a–c that when laser processing aluminum alloy in air, a large amount of heat is absorbed by the material and the material is heated and melted. The rapid cooling rate of metal materials and the limited ability of auxiliary gas blowing off make the melted materials unable to be removed in time, and they quickly solidify and accumulate in the processing area, which leads to a large roughness value. When the water velocity reaches 30 m/s (combined with Figure 7), the impact of water can compensate for the limited blowing capacity of the auxiliary gas and blow away the melt in the processing area in time, which makes the roughness of the processing area lower than that of laser processing in air.

**Figure 9:**
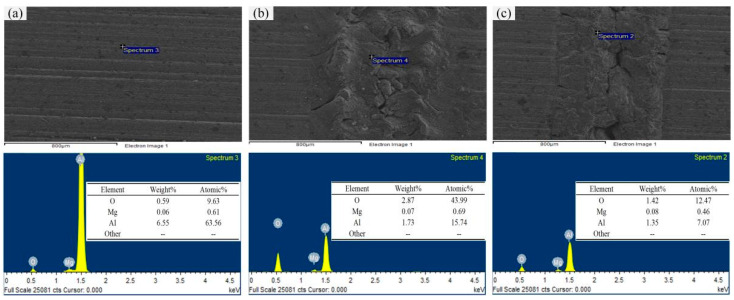
EDS analysis of aluminum alloy before and after processing. (**a**–**c**) show the elemental analyses of aluminum alloy unprocessed, laser-processed in air and laser-processed in water.

This paper further compares the changes in the main element content of the laser-processed aluminum alloy in air and water, using an X-ray energy spectrometer (EDS) to analyze the elements before and after processing. The specific measurement positions are shown in Figure 9, where Figure 9a–c show the elemental analyses of aluminum alloy unprocessed, laser-processed in air and laser-processed in water, respectively.

The original surface of the aluminum alloy material is covered with a thin oxide layer to prevent the interior of the material from being oxidized. This oxide film is mainly composed of aluminum. The laser processing in air undergoes a violent oxidation reaction and the aluminum element is consumed to generate a large amount of oxides, so the aluminum element decreases and the oxygen element increases. At high temperatures, the coefficient of action of carbon and oxygen is large, so as to form carbides and oxides that easily attract each other and aggregate. In particular, when aluminum is decomposed under laser irradiation, it can easily absorb oxygen to form alloy-rich composite alloy inclusion particles [33]. The ablation phenomenon of laser processing in water is weakened and the water can wash away the processed products in time. Excessive temperatures in the processing area will form water vapor, which will prevent the formation of oxides. At the same time, magnesium can further promote a series of reactions between aluminum and water vapor to produce Al_2_O_3_ and Al_2.667_O_4_ [34], which leads to the continuous reduction in oxygen and aluminum content.

## 4. Conclusions

In this paper, the effects of different water velocities on the depth, width and micro-morphology of the groove during the laser–water composite machining of aluminum alloys are mainly studied. The main conclusions are as follows:

(1) When the aluminum alloy material is laser-processed in air, the depth and width of the groove are 0.041 mm and 1.003 mm, respectively. At this time, the surface of the groove is seriously ablated and a large amount of slag accumulates in the groove and on both sides of the groove, which reduces the groove depth, and the processing quality is poor.

(2) During laser–water–liquid composite processing of aluminum alloy materials, the groove surface essentially has no heat-affected zone and the processing quality is better than that of laser processing in air. When the water velocity is 21 m/s, the groove depth and width reach the maximum, which are 0.125 mm and 0.974 mm, respectively. In theory, with a stronger impact force of water on the processing area, the slag produced by laser processing can be washed away in time and the depth and width of the groove will increase with the increase in water speed. However, when the water velocity increases to 30 m/s, the groove depth and width become smaller in actual processing.

(3) The surface of the original material of the workpiece is covered with a thin oxide film and the oxygen content is relatively small. When the aluminum alloy is laser-processed in air, serious ablation occurs and a large amount of slag is accumulated in the groove; the main component of the slag is Al_2_O_3_. When the aluminum alloy is laser-processed in water, the formed water vapor will prevent the formation of oxides and the magnesium element can further promote a series of reactions between aluminum and the water vapor. The main components of the product are Al_2_O_3_ and Al_2.667_O_4_.

(4) In the laser–water composite machining of aluminum alloys, water mainly plays the role of cooling and impact. The cooling of water can alleviate the phenomenon of thermal stress concentration and inhibit the generation of cracks. The impact of water can remove the slag, reduce the thickness of the recast layer and improve the processing quality. These two functions of water cause the composite processing quality to be significantly improved.

Laser–water composite machining is a promising technology to improve the machining quality of aluminum alloys.
